# Therapeutic Potential of Pien-Tze-Huang: A Review on Its Chemical Composition, Pharmacology, and Clinical Application

**DOI:** 10.3390/molecules24183274

**Published:** 2019-09-09

**Authors:** Lili Huang, Yiping Zhang, Xiaoqin Zhang, Xiuping Chen, Yitao Wang, Jinjian Lu, Mingqing Huang

**Affiliations:** 1 College of Pharmacy, Fujian University of Traditional Chinese Medicine, Fuzhou 350122, Fujian, China (L.H.) (X.Z.); 2 Engineering Innovation Center of Marine Biological Resource Development and Utilization, Third Institute of Oceanography, Ministry of Natural Resources, Xiamen 361005, China; 3 State Key Laboratory of Quality Research in Chinese Medicine, Institute of Chinese Medical Sciences, University of Macau, Macao, China (X.C.) (Y.W.)

**Keywords:** Pien-Tze-Huang, Chemical composition, Pharmacology, Clinical application, Cancer

## Abstract

Pien-Tze-Huang (PTH) is a famous and commonly used traditional Chinese medicine formula in China. It was first formulated by a royal physician of the Ming Dynasty (around 1555 AD). Recently, PTH has attracted attention worldwide due to its beneficial effects against various diseases, especially cancer. This paper systematically reviewed the up-to-date information on its chemical composition, pharmacology, and clinical application. A range of chemical compounds, mainly ginsenosides and bile acids, have been identified and quantified from PTH. Pharmacological studies indicated that PTH has beneficial effects against various cancers, hepatopathy, and ischemic stroke. Furthermore, PTH has been used clinically to treat various diseases in China, such as colorectal cancer, liver cancer, and hepatitis. In summary, PTH is a potential agent with extensive therapeutic effects for the treatment of various diseases. However, the lack of information on the side effects and toxicity of PTH is a non-negligible issue, which needs to be seriously studied in the future.

## 1. Introduction

Recently, traditional Chinese medicine (TCM) with thousands of years of clinical practice has increasingly attracted interest in the treatment of various human diseases around the world. It not only provides an alternative and promising therapeutic strategy, but also contributes a precious drug discovery source. Pien-Tze-Huang (PTH) is a well-known and precious Chinese patent medicine in China and Southeast Asian countries. It was first formulated by a royal physician of the Ming Dynasty in 1555 AD. It is composed of four TCM ingredients, including *Radix et Rhizoma Notoginseng* (Sanqi in Chinese, 85%), *Moschus* (Shexiang in Chinese, 3%), *Calculus Bovis* (Niuhuang in Chinese, 5%), and *Snake Gall* (Shedan in Chinese, 7%), which together have functions of reducing fever, detoxifying, promoting blood circulation, reducing blood stasis and swelling, and relieving pain [1]. PTH is officially used to treat acute and chronic viral hepatitis, ulcer, deep-rooted boil, traumatic injuries, and various inflammatory diseases [1,2,3]. In addition, PTH has been commonly applied to treat cancer and stroke. Its therapeutic effects on hepatocellular carcinoma and colon cancer have been recognized in clinical trials [4,5]. PTH has been categorized as one of the national treasures in the catalogue of the National Protected Traditional Chinese Medicines in 1994 attributing to its long history and significant beneficial effect against various diseases.

Although many properties of PTH have been extensively studied, a systematic and comprehensive review on its chemical, experimental, and clinical properties has not been conducted. Therefore, the aim of this review is to summarize the up-to-date progress concerning the chemical composition, pharmacology, and clinical application of PTH, thus to better understand its therapeutic potential and support its clinical application and in-depth study in the future.

## 2. Chemical Composition

PTH consists of four natural TCM materials, including *Radix et Rhizoma Notoginseng*, *Moschus*, *Calculus Bovis*, and *Snake Gall*. Many studies have been conducted to determine the main bioactive compounds of these materials. The results showed that *Radix et Rhizoma Notoginseng* mainly contains protopanaxadiol-type (e.g., notoginsenoside R1 and ginsenosides Re, Rf, Rg1, and Rg2) and protopanaxatriol-type ginsenosides (e.g., ginsenosides Rb1, Rc, Rd, and Rg3), which are considered its main bioactive compounds [6]. *Moschus* mainly contains macrocyclic ketones (e.g., muscone) and steroids (e.g., androsterone), which possess extensive biological activities [7]. *Calculus Bovis* mainly contains bile acids and their glycine or taurine conjugates, such as cholic acid, deoxycholic acid, chenodeoxycholic acid, hyodeoxycholic acid, and ursodeoxycholic acid, which are supposed to be its main characteristic and bioactive compounds [8]. *Snake Gall* mainly contains taurine-conjugated bile acids, such as taurocholic acid, taurochenodeoxycholic acid, taurodeoxycholic acid, and glycodeoxycholic acid, but their contents are markedly different from those of *Calculus Bovis*, which is the main reason for their varied efficacies [9].

**Figure 1:**
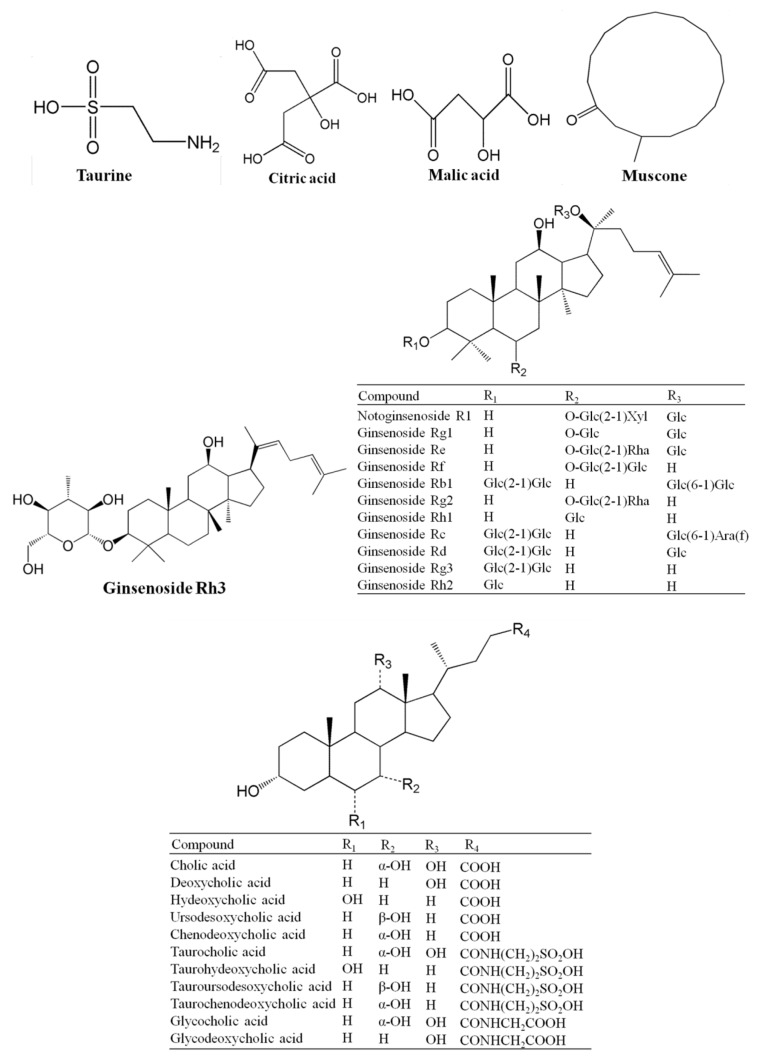
Chemical structures of 27 compounds identified from Pien-Tze-Huang (PTH).

Given the complicated influences of collection, processing, production, and storage of raw materials and products, the chemical composition of PTH is somewhat different to the chemical composition of its four components. Therefore, several analytical methods, including thin layer chromatography scanning (TLCS), high-performance liquid chromatography (HPLC), HPLC coupled with quadrupole time-of-flight mass spectrometry (HPLC-QTOF-MS), and ultra-performance LC coupled with triple quadrupole MS (UPLC-QqQ-MS), have been applied to separate and identify the chemical composition of PTH in past decades [10,11,12,13]. For example, only ginsenoside Rg1 in PTH was determined by TLCS after complicated and time-consuming alumina column purification [14], and three saponin components (notoginsenoside R1, ginsenoside Rb1, and ginsenoside Rg1) in PTH were determined by HPLC [15]. In our recent studies, 27 compounds, including taurine, citric acid, malic acid, notoginsenoside R1, ginsenosides Re, Rg1, Rf, Rg2, Rh1, Rb1, Rd, Rg3, Rc, Rh2, Rh3, cholic acid, deoxycholic acid, chenodeoxycholic acid, ursodeoxycholic acid, hyodeoxycholic acid, taurocholic acid, tauroursodeoxycholic acid, taurochenodeoxycholic acid, taurohyodesoxycholic acid, glycocholic acid, glycodeoxycholic acid, and muscone, were definitely identified in PTH by HPLC-QTOF-MS (Figure 1) [10]. Among them, 21 compounds showed significant inhibitory activities on LPS-induced TNF-α production with IC_50_ values ranging from 30.82 μM to 147.24 μM in murine mononuclear macrophage RAW 264.7 cells [10]. Thus, they were chosen as quality biomarkers of PTH, and their contents in PTH were accurately quantified by UPLC-QqQ-MS [10,11]. Furthermore, we developed a UPLC-MS/MS method to investigate the pharmacokinetic behaviors of six active compounds of PTH, including ginsenosides Re, Rg1, Rb1, and Rd, notoginsenoside R1, and muscone, after oral administration to rats and partly revealed the clinical implication of PTH in several inflammation-related diseases [13]. 

These identified compounds in PTH have been reported to have beneficial effects against various diseases, which partly reflect the therapeutic function of this formula [16,17]. For example, ginsenoside Re, one of the protopanaxadiol-type ginsenosides in *Radix et Rhizoma Notoginseng*, has been reported to exhibit potential anti-oxidant, anti-inflammatory, anticarcinogenic, and neuroprotective effects, which was well tolerated up to a 375 mg/kg/d oral dosage level in rats [18,19,20,21]. As one of protopanaxatriol-type ginsenosides in this herb, ginsenoside Rb1 has been proved to possess remarkable anti-oxidant, anti-cancer, and neuroprotective effects [22,23,24]. In addition, muscone, a volatile bioactive compound in *Moschus*, has been shown to have significant anti-inflammatory and neuroprotective effects, which exhibited liver toxicity in mice at an oral dose of 100 mg/kg [25,26,27,28]. However, more studies are still needed to determine which compounds are responsible for its therapeutic effect against different diseases. 

## 3. Pharmacology

### 3.1. Colorectal Cancer

Accumulated clinical and experimental studies have exhibited the anti-cancer properties of PTH against a broad spectrum of cancer cells, which multiply via signaling pathways. Colorectal cancer is a malignant tumor in the gastrointestinal tract, the incidence and mortality of which are both forefront worldwide [29]. In addition to its well-known anti-inflammatory activities, PTH has been shown to possess potent anti-proliferative effects against various human colorectal cancer cell lines with IC_50s_ below 1 mg/mL [30,31,32,33]. The in vivo anti-cancer potential of PTH has been recognized in different xenograft animal models [31]. PTH not only overcomes multidrug resistance (MDR) in HCT-8/5-fluorouracil (5-FU) cells but also attenuates 5-FU-induced intestinal mucositis in CT-26 tumor-bearing xenograft mice [34,35,36]. Furthermore, it exhibits synergistic effects when combined with chemotherapy drugs, such as fluorouracil, oxaliplatin, and capecitabine, in clinical in China [37,38]. However, the molecular mechanism underlying its anti-cancer effect remains unclear.

The induction of cell cycle arrest and apoptosis is suggested to be the primary reason for the anti-proliferative effect of PTH. PTH inhibits the proliferation of Caco-2 and HCT-8 cells by inducing G1/S-phase arrest, which may be due to the inhibition of cyclin-dependent kinase (CDK)4, CDK6, Cyclin D1, and c-Myc [32,33]. PTH-induced cell cycle arrest is often accompanied by apoptosis. These two actions are at least partially mediated by inhibiting interleukin-6 (IL-6)/signal transducer and activator of transcription 3 (STAT3) pathway and its target genes, such as suppressor of cytokine signaling 3 (SOCS3) [31,39]. Moreover, the inhibition of the IL-6/STAT3 pathway has been suggested to account for the beneficial effects of PTH in the treatment of ulcerative colitis [40]. As one of the MiR-34 family, miR-34c exhibits anti-proliferative and pro-apoptotic functions. Wan et al. (2017) showed that PTH inhibits proliferation and induces apoptosis in HCT-8 cells partially by upregulating miR-34c-5p expression, which may provide a novel perspective for investigating its mechanisms.

Metastasis is a complicated process related to the spread of malignant cancer cells from a primary position to another position. In HCT-8 cells, PTH inhibits migration and invasion in a dose-dependent manner by decreasing the expressions of transforming growth factor β1 (TGF-β1), Smad2/3, Smad4, zinc finger E-box binding homeobox 1 (ZEB1), and ZEB2 and upregulating the expressions of miR-200a, miR-200b, and miR-200c [41]. In an orthotopic liver metastasis model of colorectal cancer, PTH exhibits beneficial effects against metastasis without significant toxicity. Furthermore, it inhibits the expressions of TGF-β, Smad2/3, p-Smad2/3, and Smad4 in tumor tissues, suggesting its inhibitive activities on the TGF-β/Smad pathway [42]. It also inhibits hypoxia-induced epithelial–mesenchymal transition (EMT) in HCT-8 cells through suppressing hypoxia-inducible factor 1 (HIF-1) pathway, which may be another important molecular mechanism [43]. A similar effect has also been found in HCT-8/5-FU cells [34]. Lymphangiogenesis, the production of new lymphatic vessels, plays a crucial role in tumor development, especially in cancer metastasis. Vascular endothelial growth factor-C (VEGF-C) is important in cancer metastasis as well. A recent study indicated that PTH reduces cell migration and VEGF-C expression in various colorectal cancer cell lines, and inhibits lymphangiogenesis by reducing cell migration and tube formation in a VEGF-C-stimulated human lymphatic endothelial cell model, indicated that inhibiting VEGF-C may be one of its potential molecular mechanisms [44].

Angiogenesis plays a critical role in tumor progress, which is one of the common therapeutic strategies in cancer treatment. An in vitro study showed that PTH inhibits migration and tube formation of human umbilical vein endothelial cells (HUVECs) in a dose-dependent manner without affecting their viability. Furthermore, PTH inhibits hypoxia-induced activation of HIF-1α and decreases VEGF-A and vascular endothelial growth factor receptor 2 (VEGFR2) expressions in both HT-29 and HUVECs [45]. In a colorectal cancer mouse xenograft model, PTH decreases tumor volume, tumor weight, microvessel density in tumor tissues; and the expressions of angiogenic factors, including iNOS, eNOS, VEGFR2, VEGF-A, bFGF, and basic fibroblast growth factor (bFGFR) [46]. However, more studies are needed to elucidate the anti-angiogenesis effect and molecular mechanisms of PTH.

Several studies demonstrated that PTH may be a potential multidrug resistance (MDR)-reversing drug that restores the sensitivity of MDR cancer cells [34,35,36]. PTH dose-dependently inhibits the proliferation of HCT-8/5-FU cells, increases the intercellular accumulation of Rhodamine-123, and downregulates ATP-binding cassette subfamily C member 2 (ABCC2) expressions in HCT-8/5-FU cells, indicating its ability to overcome MDR. Moreover, PTH inhibits MDR/EMT-enhanced migration and invasion capabilities and suppresses MDR-induced activation of TGF-β signaling in HCT-8/5-FU cells [34]. Another recent study also found that PTH inhibits proliferation and induces apoptosis of HCT-8/5-FU cells by regulating the expressions of CDK4, cyclin D1, B-cell lymphoma 2/Bcl-2-associated X protein (Bcl-2/Bax) ratio, caspase-9, and caspase-3, which may be mediated by modulating the miR-22/c-Myc signaling network [35]. Notably, PTH not only overcomes MDR but also attenuates chemotherapy drug-induced side effects. In CT-26 tumor-bearing xenograft mice, PTH improves 5-FU-induced diarrhea and intestinal pathological damages without significant effect on body weight loss, which is partly related to its anti-apoptotic effect in the intestinal crypt [36].

Cancer stem cells (CSCs) play a crucial role in tumor development and drug resistance, suggesting that targeting CSCs may be a promising approach for cancer treatment. In the side population (SP) isolated from HT-29 cells, PTH dose-dependently reduces the percentage of SP cells, inhibits their proliferation and sphere-forming capacity, and inhibits ABCB1 and ABCG2 levels, indicating that the inhibition of CSCs may be a potential mechanism of PTH [47]. A similar study by Qi et al. demonstrated that PTH dose-dependently inhibits proliferation and induces apoptosis and differentiation of SP cells isolated from SW480 cells. In addition, PTH treatment inhibits the mRNA and protein expressions of hairy and enhancer of split 1 (Hes1) and Notch1 of SP cells [48].

### 3.2. Liver Cancer

Previous experimental and clinical studies have exhibited promising therapeutic effects in the treatment of liver cancer. In human BEL-7402 hepatocellular carcinoma cell lines, PTH inhibits proliferation by inhibiting cyclin D1 and CDK4 expressions and induces apoptosis by decreasing Bcl-2/Bax ratio, which may be due to the upregulation of tumor suppressor miR-16 [49]. Similar anti-proliferative and anti-apoptotic effects have also been observed in human SMMC-7721 and HepG2 CSCs [50].

### 3.3. Osteosarcoma

Osteosarcoma is the most common form of primary bone cancer that usually occurs in teenagers or young adults. An in vitro study has shown that PTH dose-dependently inhibits the viability of MG-63 cells by inducing apoptosis, which may be mediated by downregulating p21, p53, Bcl-xl, Bcl-2, PI3K, p-Akt, and p-ERK1/2 levels and upregulating caspase-3, caspase-9, Bax, and cleaved-PARP levels [51]. In human osteosarcoma-bearing nude mice, PTH treatment alone or in combination with p27 gene recombinant adenovirus exhibited significant tumor growth inhibition effects (34.1% and 63.8%, respectively), suggesting that both treatments are beneficial for osteosarcoma treatment [52]. Moreover, PTH dose- and time-dependently inhibits the viability of U2OS/ADM cells through inducing G2/M-phase cell arrest and apoptosis, which may be mediated by the downregulation of Bcl-2, survivin, and P-gp and the upregulation of Bax [53].

### 3.4. Other Cancers

In addition to the abovementioned cancer types, PTH has been used in the treatment of other cancers, including neuroblastoma, ovarian cancer, and breast cancer, with similar mechanisms. The anti-proliferative activities of PTH in vitro are summarized in Table 1. A previous study showed that PTH can dose-dependently inhibit the survival of neuroblastoma SH-SY5Y cells without affecting normal fibroblast NIH-3T3 cells [54]. PTH also inhibits the viability of human ovarian cancer OVCAR-3 cell line by inducing G1/S-phase cell arrest without inducing apoptosis. This effect is at least partially mediated by inhibiting Akt–mammalian target of rapamycin (mTOR) pathway [55]. In human breast cancer MCF-7/ADR cells, PTH dose-dependently inhibits their proliferation, increases the intercellular accumulation of adriamycin, and reduces ABCG2 and ABCB1 expressions. Moreover, PTH inhibits EMT, migration, and invasion and suppresses the activation of TGF-β1 in MCF-7/adriamycin cells [56]. Further experimental and clinical studies are needed to clearly define these effects.

### 3.5. Hepatopathy

Accumulated evidence demonstrated that PTH can be potentially used in treating various hepatic diseases, such as viral hepatitis, alcoholic hepatitis, and fatty liver. Previous studies have shown that PTH protects against liver damage, decreases alanine aminotransferase (ALT) and aspartate aminotransferase (AST) levels in a mouse with acute hepatitis induced by carbon tetrachloride or galactosamine, and upregulates the mRNA expressions of anti-microbial protein 1 (AP1) and nuclear factor kappa-B (NF-κB) in hepatoma cells [2]. Moreover, PTH exhibits similar hepatoprotective effect in alcoholic and non-alcoholic fatty liver animal models. In alcoholic and high-fat diet-induced alcoholic fatty liver rats, PTH improves hepatic function defects, hepatic pathology, and impairment in lipid metabolism by inhibiting the homocysteine-related protein kinase RNA-like endoplasmic reticulum kinase (PERK)/eukaryotic translation initiation factor 2α (eIF2α) pathway [57]. In high-fat diet-induced non-alcoholic fatty liver rats, PTH improves liver function and lowers blood lipid, which may be related to regulating the farnesoid X receptor (FXR)/small heterodimer partner (SHP)/sterol regulatory element binding protein-1c (SREBP-1) pathway [58].

### 3.6. Ischemic Stroke

Stroke is one of the major causes of death and disability worldwide, and more than 80% of cases are categorized as ischemic stroke. Increasing evidence suggest that PTH is a potential protective and therapeutic agent for ischemic stroke. In spontaneously hypertensive rat (SHR) and stroke-prone SHR, PTH significantly reduces cell death in the hippocampus and cerebellum caused by chronic ischemia and hypertensive stroke possibly by inhibiting cell apoptosis or ROS/oxidative damage in the mitochondria [59]. Moreover, PTH can obviously make the stroke less serious and notably postpone death after its occurrence [59,60].

In our previous study, the transient middle cerebral artery occlusion (tMCAO) model was used to investigate the effect of PTH on cerebral ischemia/reperfusion injury. The results showed that PTH significantly improves neurological deficit infarct volume, and downregulates the levels of IL-1β, IL-6, and TNF-α in brain tissue of tMCAO rats. Furthermore, PTH decreases the cytosolic levels of cytochrome C, p53, Bax, cleaved caspase-3, and cleaved caspase-9 and increases the mitochondrial levels of cytochrome C and Bcl-xl and the phosphorylation levels of Akt and GSK-3β, indicating that inhibiting mitochondria-mediated neuronal apoptosis may be one of its important mechanisms [61]. Although much progress has been made in experimental studies, more clinical studies are needed to verify these effects. The pharmacological activities and mechanisms of PTH in vivo have been summarized in Table 2.

Notably, PTH was simultaneously used to treat cancer and stroke in different studies, whereas mechanistically, it has a pro-apoptotic effect in cancer and anti-apoptotic effect in stroke [31,61]. In general, the same drug or therapy can be used to treat different diseases and is considered to be one of the main characteristics of TCM. Besides, various results could be obtained due to different disease pathogenesis as well as diverse dosage regimens. However, the detailed molecular mechanisms are required for further study. 

## 4. Clinical Application

### 4.1. Colorectal Cancer

In past decades, the clinical efficacy of PTH on colorectal cancer has been investigated in China. As early as 1993, PTH alone has been observed to improve clinical symptoms and life quality in a preliminary clinical observation containing 25 advanced colorectal cancer patients [5]. In another clinical observation containing 47 advanced colorectal cancer patients, PTH in combination with 5-FU, oxaliplatin, and leucovorin exhibited better short-term efficacy, reduced the toxicity and side effects of chemotherapy, and improved the life qualities of advanced colorectal cancer patients compared with single chemotherapy [37]. A similar synergistic effect of PTH combined with oxaliplatin and capecitabine was observed in another clinical observation containing 68 advanced colorectal cancer patients [38]. However, more restrictive clinical trials are needed to further confirm its efficacy in colorectal cancer treatment.

### 4.2. Liver Cancer

Several clinical studies on liver cancer have been conducted in the past decades. In a 1994 clinical observation containing 42 patients with moderate or advanced liver cancer, PTH combined with conventional therapies achieved lower exacerbation rate and improved clinical symptoms and life quality compared with single chemotherapy [62]. In another randomized, double-blind, placebo-controlled clinical trial that enrolled 207 patients with primary liver cancer, PTH in combination with interventional chemotherapy exerted significant improvements in reducing tumor size, improving life quality, relieving pain, and reducing the toxicity and side effects of chemotherapy compared with single interventional chemotherapy [63]. Intriguingly, PTH can improve weakened immunity inhibited by transcatheter arterial chemoembolization in 40 patients with primary liver cancer [64].

### 4.3. Hepatitis

A recent clinical observation demonstrated that PTH can improve clinical symptoms and signs, alleviate digestive tract symptoms, protect liver function, and decrease the levels of HBV-DNA and HbeAg in 47 patients with chronic hepatitis B, with an efficacy similar to or better than polyene phosphatidylcholine [65].

### 4.4. Other Diseases

PTH has also been applied in treating other diseases, including ulcers and phlebitis. Two clinical observations have verified that PTH can accelerate the healing of recurrent aphthous ulcer and decrease the risk of recurrence without an increase in obvious adverse reactions [66,67]. Moreover, in a clinical study containing 31 patients with phlebitis induced by amiodarone, PTH exhibited better clinical efficacy than magnesium sulfate [68]. 

Except for a well-controlled clinical study by Zhao et al. [63], most of above studies were preliminary clinical observation. Furthermore, most of studies were focused on the combination of PTH and routine therapy but not the single PTH. Clinical application of PTH on various diseases has been summarized in Table 3.

## 5. Conclusions and Prospects

This review mainly summarizes the up-to-date progress concerning the chemical composition, pharmacology, and clinical application of PTH. The findings indicate that PTH is a safe and effective therapeutic agent against various diseases. In the past decades, researchers have focused on the pharmacological activities of PTH, particularly on its anti-cancer effects. Despite the great progress in the study of PTH, several points still need to be noted for better understanding and utilization of this traditional formula. First, although PTH has been increasingly used to treat various diseases in China and Southeast Asian countries, especially cancer, only few relevant randomized, double-blind, placebo-control, and multi-center clinical trials exist. Therefore, more restrictive clinical trials are needed to evaluate the safety and efficacy of PTH alone or in combination with routine therapy. Second, in spite of no side effects or toxicity reported in the peer reviewed papers, we believe that the potential side effects or toxicity of PTH still need to be seriously studied in future research, and more attention should be paid to its misuse, overdose, long-term administration, or unreasonable drug combination. Third, more pharmacokinetic studies are needed to illuminate the absorption, distribution, metabolism, and excretion of PTH, which can better guide its clinical application. Fourth, the specific compounds responsible for the beneficial effects of PTH on different diseases, as well as the action of mechanism, still remain to be elucidated. Fifth, the quality control of PTH is still important for guaranteeing the safety and efficacy of its preparation, though some analysis methods have made significant contributions. Future studies should focus on the collection, processing, production, and storage of its raw materials and finished products. Finally, most of the research on PTH are from China currently, and it needs more and more abroad scientists’ participation. Collectively, finding of presenting studies highlight the therapeutic potential of PTH in various diseases, especially cancer.

## Figures and Tables

**Table 1:** The anti-proliferative activities of Pien-Tze-Huang (PTH) in vitro.

Cancer Types	Cell Lines	IC_50_	Reference
Colorectal cancer	HT-29	0.65 mg/mL/24 h	[30]
	Caco-2	>1 mg/mL/24 h	[26]
	CT-26	0.75 mg/mL/24 h	[42]
	HCT-8	0.25-0.5 mg/mL/6 h	[43]
		0.25-0.5 mg/mL/24 h	[41]
	HCT-8/5-Fu	>0.25 mg/mL/48 h	[34]
	SW480 SP	0.75 mg/mL/48 h	[48]
	HCT-116	0.75 mg/mL/24 h	[44]
		0.5-0.75 mg/mL/48 h	[44]
	SW620	<0.75 mg/mL/24 h	[44]
		>0.5 mg/mL/48 h	[44]
Neuroblastoma	SH-SY5Y	<400 μg/mL/24 h	[54]
	NIH-3T3	>400 μg/mL/24 h	[54]
Osteosarcoma	U2OS	1.2 mg/mL/48 h	[53]
	U2OS/ADM	>1.2 mg/mL/24 h	[53]
		<1.2 mg/mL/48 h	[53]
		0.8-1.2 mg/mL/72 h	[53]
	MG-63	500-750 μg/mL/24 h	[51]
Ovarian cancer	OVCAR-3	>1000 mg/mL/24 h	[55]
Liver cancer	BEL-7402	>0.75 mg/mL/48 h	[49]
		0.5-0.57 mg/mL/72 h	[49]
Breast cancer	MCF-7/ADM	>0.75 mg/mL/48 h	[56]

Note: IC_50_, half maximal inhibitory concentration; 5-Fu, fluorouracil; SP, side population; ADM, adriamycin.

**Table 2:** The pharmacological activities and mechanisms of PTH in vivo.

Disease Type	Animal Model	Method	Effects	Mechanisms	Reference
Colorectal cancer	HT-29 tumor-bearing xenograft mice	234 mg/kg/d, 5 days a week for 16 days	reduced tumor volume and tumor weight without apparent adverse effect, inhibited tumor cell proliferation, promoted apoptosis	↑Bax, Bax/Bcl-2; ↓Cyclin D1, CDK4, Bcl-2, p-STAT3	[31]
	CT-26 tumor-bearing xenograft mice	250 mg/kg/d for 4 days	alleviated the severity of 5-FU-induced diarrhea and morphological intestinal damages, inhibited cell apoptosis in the intestinal crypt	↑Bax, Bax/Bcl-2; ↓Bcl-2,	[36]
	HT-29 tumor-bearing xenograft mice	234 mg/kg/d, 5 days a week for 16 days	suppressed tumor volume and weight, inhibited tumor angiogenesis	↓p-STAT3, p-ERK, p-Akt, p-JNK, p-p38, VEGF-A, VEGFR2, bFGF, bFGFR, iNOS, eNOS	[46]
	CT-26 cells liver metastasis animal model in athymic male nude mice	234 mg/kg/d for 14 days	inhibited tumor liver metastasis without apparent toxicity, inhibited EMT	↑E-cadherin; ↓N-cadherin, TGF-β, p-Smad2/3, Smad4	[42]
Osteosarcoma	Saos-2 tumor-bearing xenograft mice	0.234 mg/g twice daily for 6 weeks combined with p27 gene once for 3 days	inhibited tumor growth	↑p27	[52]
Hepatic diseases	acute hepatitis mouse induced by carbon tetrachloride	0.5 g/kg three times over 36 h	ameliorated hepatic pathology	↑AP1, NF-κB; ↓ALT	[2]
	alcohol and high-fat diet rats	0.5, 1, and 2g/kg/d for 3 weeks	ameliorated the defects in hepatic function, hepatic pathology and the impairment in lipid metabolism	↓GRP78, GRP9, p-eIF2a	[57]
	nonalcoholic fatty liver rats	0. 5, 1.0, 2.0 g/kg/d for 4 weeks	improved liver function, lowered blood lipid	↑SREBP-1c mRNA expression; ↓FXR, SHP mRNA expression	[58]
Ischemic stroke	SHR rats and stroke prone SHR rats	18 mg/kg/d for 3 months	reduced cell death in hippocampus and cerebellum by chronic ischemia and hypertensive stroke	↓QCR_2_, cleaved caspase-3	[59]
	MCAO rats	180 mg/kg/d for 4 days	reduced cerebral infarct volume, improved neurological deficit, attenuated inflammatory response, inhibited neuronal apoptosis	↑NeuN, cytosolic Cyt C, Bax, P53,cleaved caspase-3; ↓IL-1β, IL-6, TNF-α, Bcl-xl, p-AKT, p-GSK-3β, caspase-9, mitochondrial Cyt C	[61]

Note:↑, upregulation; ↓, downregulation; CRC, colorectal cancer; Bcl-2, B cell lymphoma/lewkmia-2; Bax, Bcl-2 associated X protein; CDK, cyclin-dependent kinase; p-STAT3, phosphor-signal transducer and activator of transcription 3; p-ERK, phosphor-endoplasmic reticulum kinase; p-JNK, phosphor-c-Jun N-terminal kinase; VEGF-A, vascular endothelial growth factor-A; VEGFR2, vascular endothelial growth factor receptor 2; bFGF, basic fibroblast growth factor; bFGFR, basic fibroblast growth factor receptor; iNOS, inducible nitric oxide synthase; eNOS, endothelial nitric oxide synthase; TGF-β1, transforming growth factor-β1; Smad, drosophila mothers against decapentaplegic protein; AP1, anti-microbial protein 1; NF-κB, nuclear factor kappa-B; ALT, alanine aminotransferase; GRP, glucose-regulated protein; p-eIF2α, phosphor-eukaryotic translation initiation factor 2α; SREBP-1c, sterol regulatory element binding protein-1c; FXR, farnesoid X receptor; SHP, small heterodimer partner; SHR, spontaneously hypertensive rat; QCR2, Cytochrome b-c1 complex subunit 2; caspase-3, cysteine aspartic protease 3; MCAO, middle cerebral artery occlusion; NeuN, nuclear antigen neuron; IL-1β, interleukin-1β; IL-6: interleukin-6; TNF-α, tumor necrosis factor α; p-GSK-3β, phosphor-glycogen synthase kinase 3β; caspase-9, cysteine aspartic protease 9; Cyt C, cytochrome C.

**Table 3:** Clinical application of PTH on various diseases.

Disease Type	Dose and Course of Treatment	Combined Medication	PTH/Control (n)	Efficacy	Side Effects	Reference
Advanced colorectal cancer	1.8 g/d, p.o., 28 days	-	25/0	improved clinical symptoms and life quality	N/A	[5]
	1.2 g/d, p.o., 24 weeks	5-FU (300 mg/m^2^/d, i.v., 2 days), oxaliplatin (85 mg/m^2^/d, i.v., 2 days), leucovorin (200 mg/m^2^/d, i.v., 1 day)	24/23	had better short-term efficacy, reduced the toxicity and side effects of chemotherapy, and improved life qualities	N/A	[38]
	1.2 g/d, p.o., 24 weeks	oxaliplatin (130 mg/m^2^/d, i.v., 1 day) and capecitabine (1000 mg/m^2^/d, p.o.)	34/34	improved life quality, reduced the toxicity and side effects of chemotherapy	N/A	[37]
Moderate or advanced liver cancer	1.0 g/d, p.o.,30 days	routine radiotherapy or chemotherapy	42/0	achieved lower exacerbation rate, improved clinical symptoms and life quality compared with single chemotherapy	N/A	[62]
Primary liver cancer	1.8 g/d, p.o.,56 days	routine interventional chemotherapy	102/105	reduced tumor size, improved life quality, relieved pain, reduced the toxicity and side effects of chemotherapy	N/A	[63]
	1.8 g/d, p.o.,56 days	transcatheter arterial chemoembolization	20/20	increased NK, CD_3_, CD_4_ and CD_4_/CD_8_ levels	N/A	[64]
Chronic hepatitis B	1.8 g/d, p.o., 3 months	polyenephosphatidylcholine (1368 mg/d, p.o.)	47/47	improved digestive tract symptoms, protected liver function	N/A	[65]
Recurrent aphthous ulcer	1.5 g/d, p.o.	Niuhuang Jiedu Pills (2.43 g/d, p.o.)	31/35	accelerated the healing of recurrent aphthous ulcer, decreased the risk of recurrence	N/A	[66]
	2.13 g/d, p.o.	Niuhuang Jiedu Pills (2.13 g/d, p.o.)	37/37	accelerated the healing of recurrent aphthous ulcer, decreased the risk of recurrence	N/A	[67]
Phlebitis induced by amiodarone	0.75 g/d, external treatment, 4 days	50% magnesium sulfate	31/31	improved redness, swelling, heat, pain	N/A	[68]

Note: PTH, the PTH-treated group; Control, the control group; NK, natural killer cell; CD, cluster of differentiation; N/A, not available.
